# Supplementary figures and images for: Lateralised memory networks may explain the use of higher-order visual features in navigating insects
Source: PLoS Comput Biol. 2025 Jun 23;21(6):e1012670. doi: 10.1371/journal.pcbi.1012670 (PMC12225813; doi:10.1371/journal.pcbi.1012670)

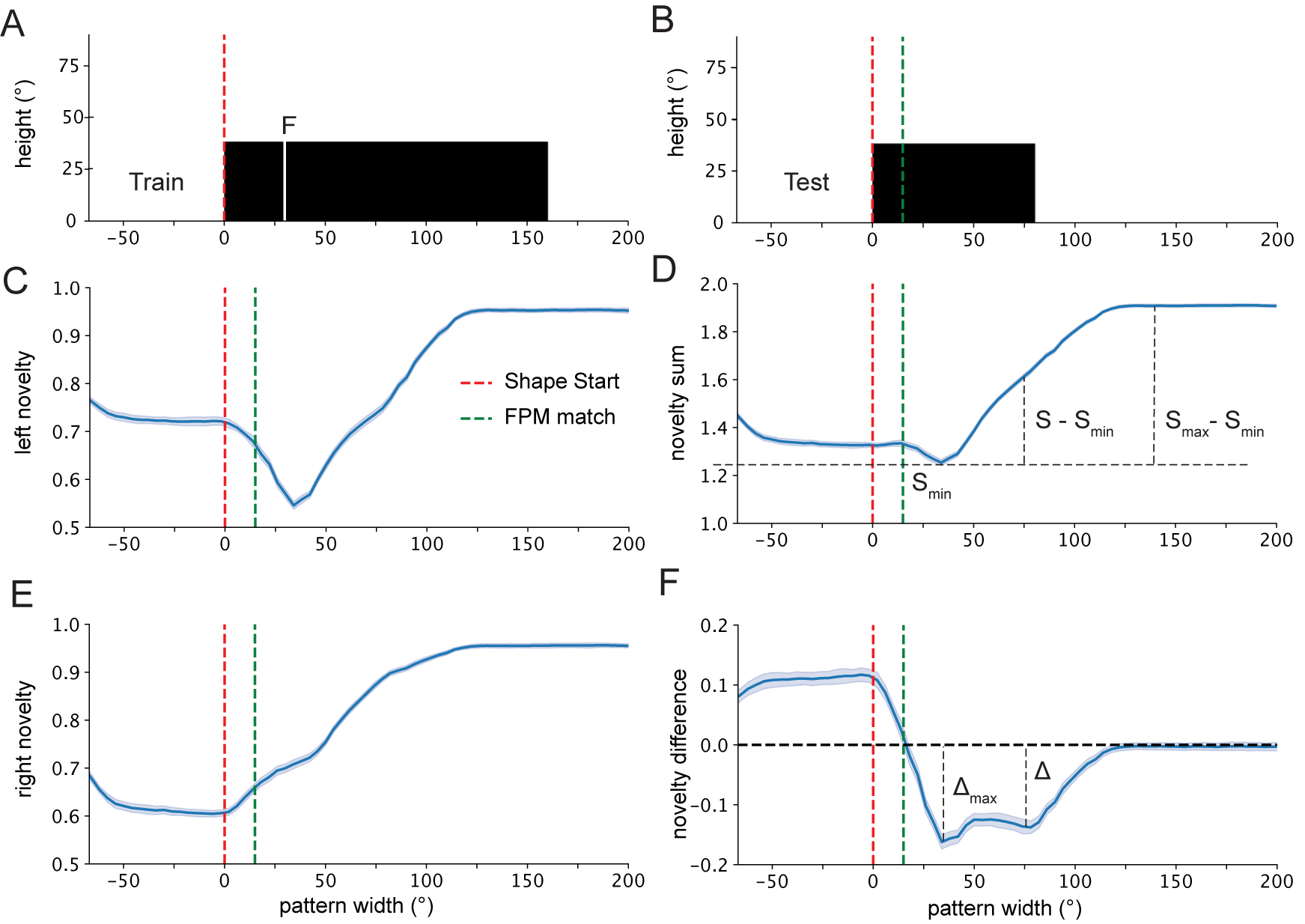

Supplement: S1 Fig — (A) The train image used for this example is a rectangle of width 160∘ and height 38∘ with feeder inset 30∘ from the left side of shape (Fig 2A). (B) The test shape is a rectangle of width 80∘ and height 38∘ (Fig 2B). (C) The left novelty is plotted as a function of angular orientation, with 0∘ being the direction to the left edge of the train and test shapes. (D) The sum of left and right novelties is plotted. Smin denotes the minimum value of the signal. Smax denotes the maximum value of the signal. S denotes the value of the signal at an angular orientation of 75∘. (E) The right novelty is plotted as a function of angular orientation. (F) The difference of left and right novelties is plotted against angular orientation. Δmax denotes the maximum novelty difference in absolute value. Δ denotes the novelty difference at an angular orientation of 75∘. (TIF) [file pcbi.1012670.s001.tif]

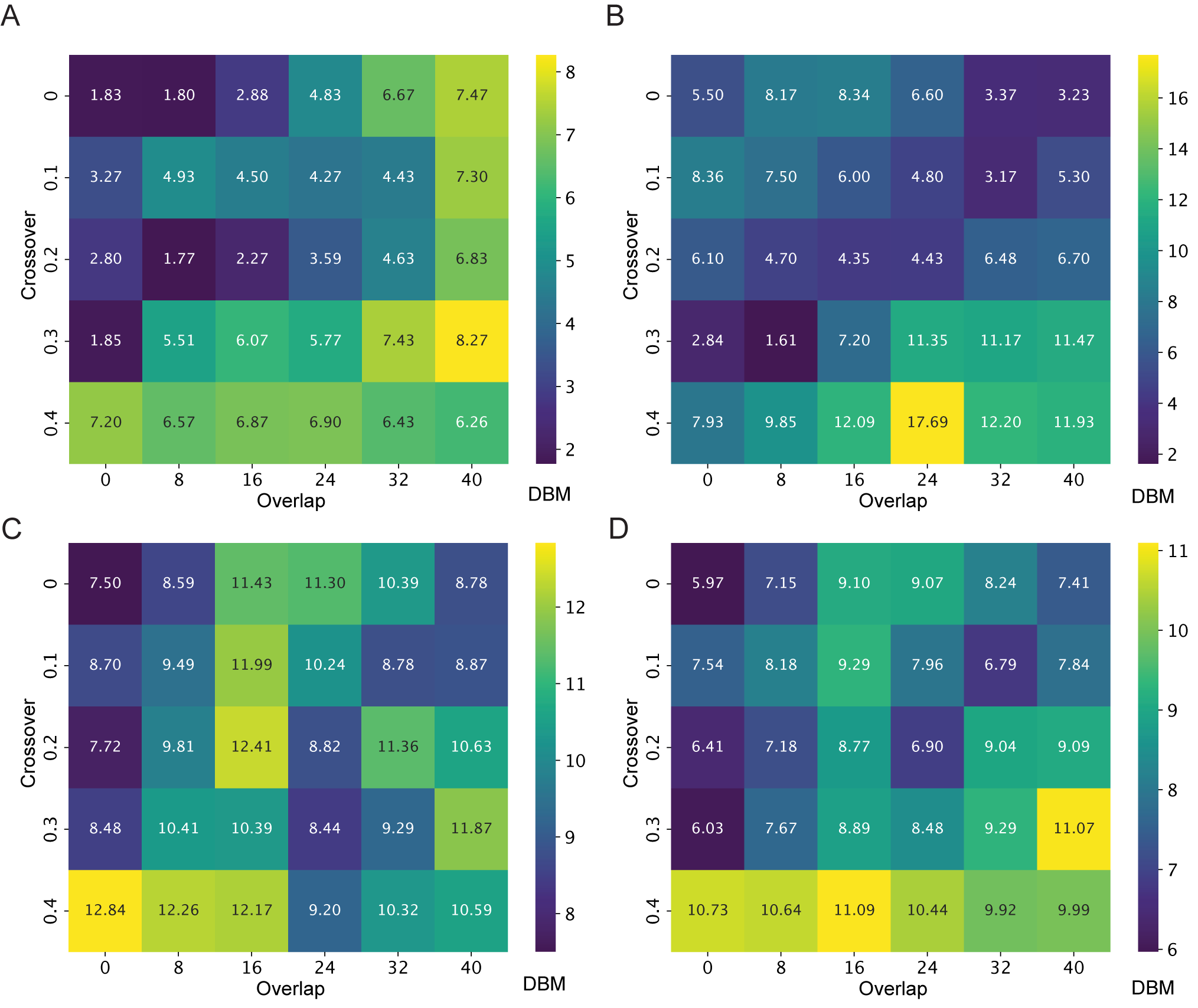

Supplement: S2 Fig — Heatmaps of average performance of the model, denoted as DBM (“distance between modes”) for all combinations of crossover [0, 0.1, 0.2, 0.3, 0.4] and overlap [0∘, 8∘, 16∘, 24∘, 32∘, 40∘] parameter values shown for (A) First set of experiments. (B) Second set of experiments. (C) Third set of experiments. (D) Overall. (TIF) [file pcbi.1012670.s002.tif]

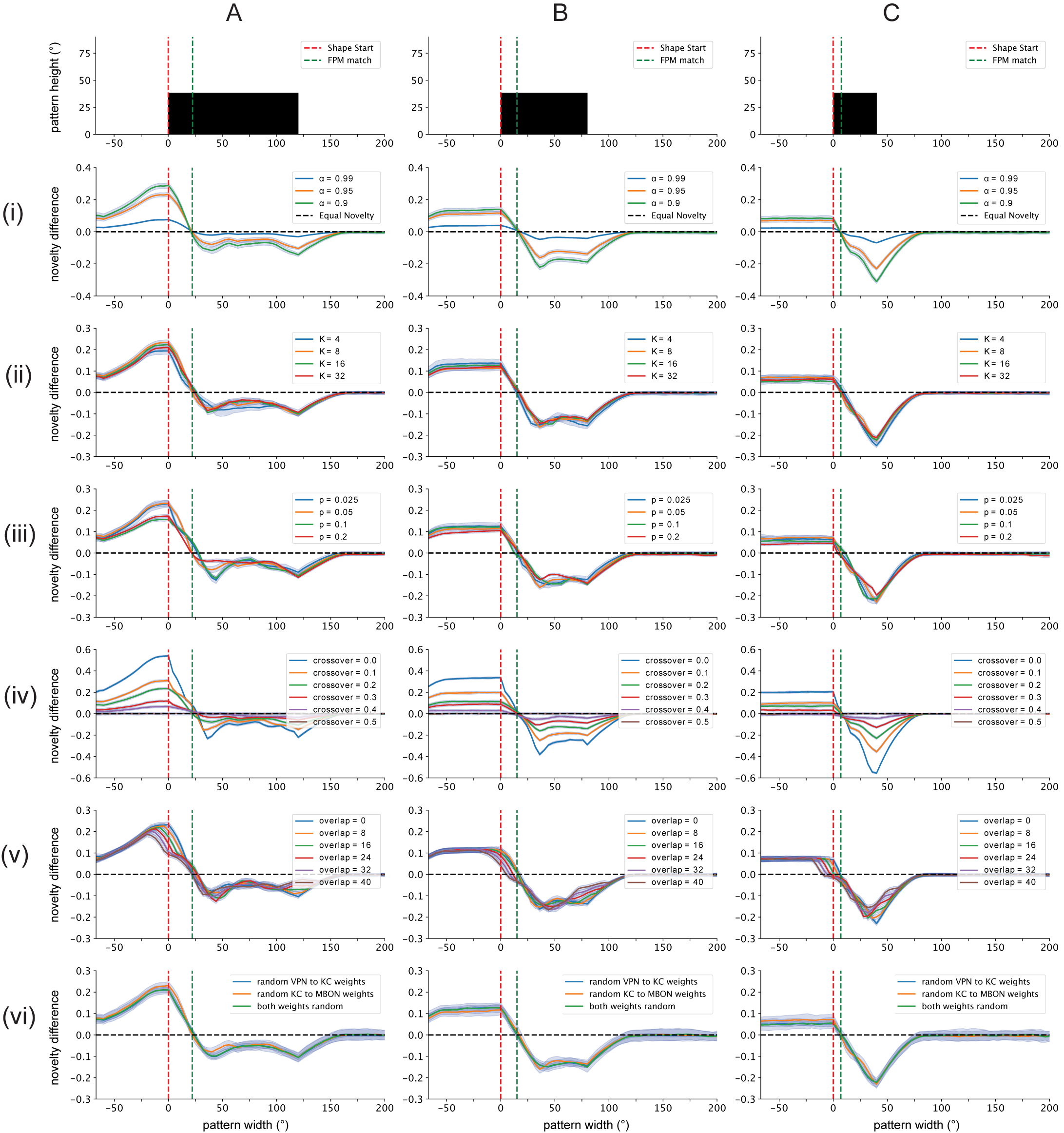

Supplement: S3 Fig — The train image used for all results in this figure is a rectangle of width 160∘ and height 38∘. Column (A) consists of testing on a 120∘ by 38∘ rectangle. Column (B) consists of testing on a 80∘ by 38∘ rectangle. Column (C) consists of testing on a 40∘ by 38∘ rectangle. Row (i) consists of rotational novelty difference plots for different values of parameter α in the range [0.9, 0.95, 0.99]. Row (ii) studies parameter K in the range [4, 8, 16, 32]. Row (iii) studies parameter p in the range [0.025, 0.05, 0.1, 0.2]. Row (iv) studies parameter crossover in the range [0, 0.1, 0.2, 0.3, 0.4, 0.5]. Row (v) studies parameter overlap in the range [0∘, 8∘, 16∘, 24∘, 32∘, 40∘]. Row (vi) studies the model with randomly initialised connection weights. In the first case we initialise vPN to KC weights uniformly in [0,2/K]. In the second case KC to MBON weights are uniformly distributed in [0,2/pNKC]. In the third case, both are initialised randomly as described above. (TIF) [file pcbi.1012670.s003.tif]

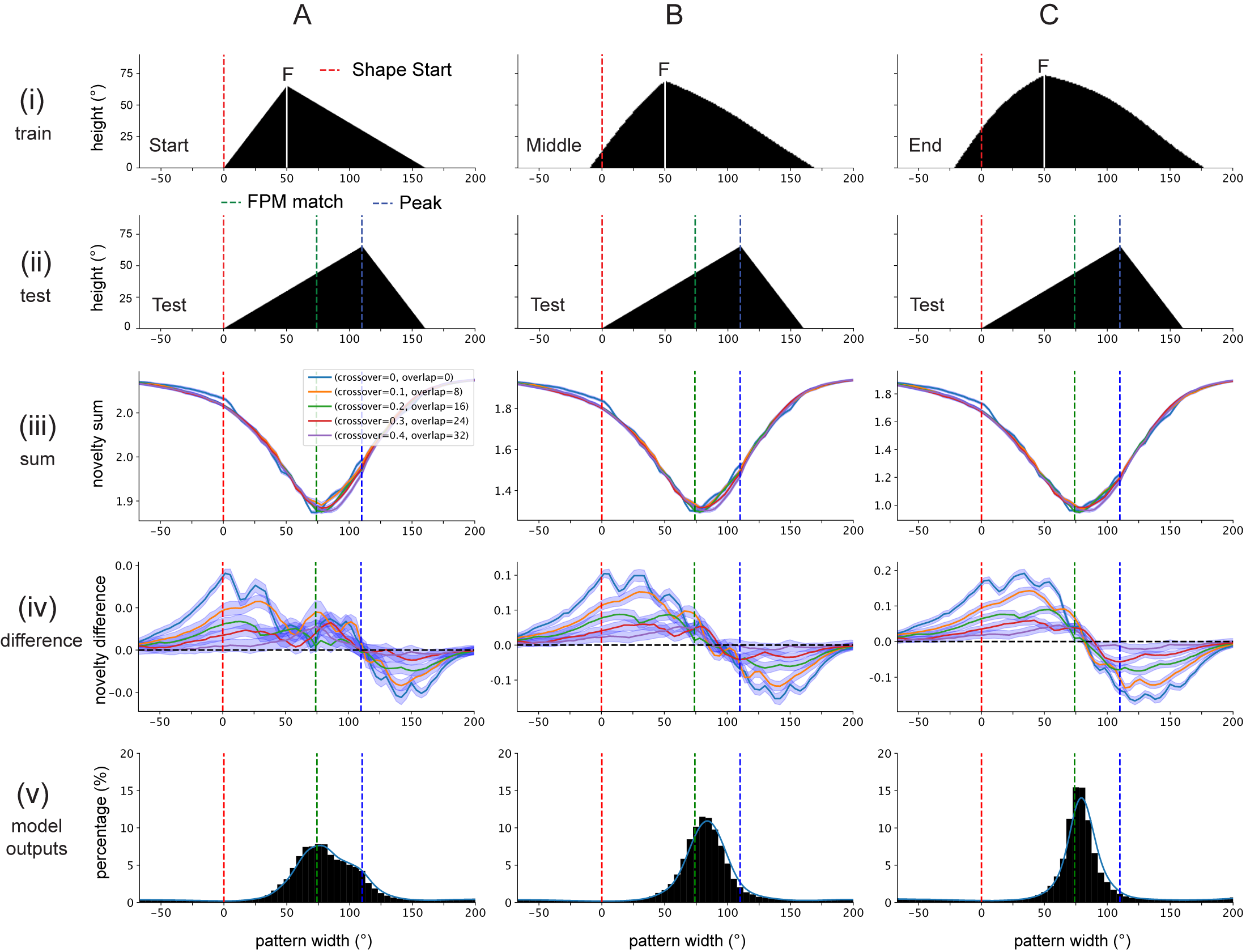

Supplement: S4 Fig — (A) Training only on first view (as experienced from centre of the arena). (B) Training up until halfway to the feeder location (Ntrain = 15). (C) Training on the whole path (Ntrain = 30). (i) The training shape as it is experienced from the last training location. (ii) The test shape. (iii) The novelty sum output of the trained model after testing. We display the curves for 5 different parameter settings (crossover, overlap = [0,0∘], [0.1, 8∘], [0.2, 16∘], [0.3, 24∘], [0.4, 32∘]) (iv) The novelty difference output of the model. (v) The estimated distributions (pooled) for all three modes of training. (TIF) [file pcbi.1012670.s004.tif]
