# Supplementary material for: Lateralised memory networks may explain the use of higher-order visual features in navigating insects
Source: PLoS Comput Biol. 2025 Jun 23;21(6):e1012670. doi: 10.1371/journal.pcbi.1012670 (PMC12225813; doi:10.1371/journal.pcbi.1012670)
Supplement: S1 Appendix — We include additional mathematical derivations supporting the statements made in the second section of the results. (PDF) [file pcbi.1012670.s005.pdf]

Additional Derivations

1. INTRODUCTION

This document contains additional derivations supporting the statements made in Section 2 of the Main Text (Results). The key purpose of this section is to show that the left and right novelty outputs of a (lateralised) Mushroom Body model equate approximately when train and test images are aligned at the same FPM value. We make these statements more explicit in this supplemental document.

2. MAIN TEXT RESULTS

As with the Main Text, we consider the case where the test shape can be contained within the train shape separating the (hemispheric) shape overlap regions into 4 areas: train-left (X), test-left (Y), train-right (W), test-right (Z). In the Main Text, we show how having train and test shapes aligned at the same FPM value is equivalent to having equal covering proportions in left and right hemispheres.

$$\gamma_L = \frac{Y}{X} = \frac{Z}{W} = \gamma_R \tag{S1}$$

Assuming that cells are identically distributed and fire with probability p overall, we also showed how the novelty (in left or right hemispheres) can be written as a function of the conditional firing probability of a single cell.

$$\mathbb{E}\left[\text{Novelty L/R}\right] = A - B \times pN_{KC} \times \mathbb{P}\left(\text{KC}^{\text{Test}}_{\text{L/R}} = 1 \mid \text{KC}^{\text{Train}}_{\text{L/R}} = 1\right) \tag{S2}$$

The previous equation implies that left and right novelties equate if the conditional firing probabilities (for a single cell) equate in left and right hemispheres. The additional step required to link these two results together is to show how the conditional firing probabilities equate across hemispheres approximately where $\gamma_L = \gamma_R$.

3. ADDITIONAL RESULTS

We start with studying solely the left hemisphere, but the methods used carry over to a study of the right hemisphere. The key property that determines if a Kenyon Cell fires is that it needs a number of coincidental connections (to firing vPNs) that is significantly above the population average. Suppose there are T vPNs in total, with X of them firing in response to the train image. Because the pattern of connections of a KC is assumed to be random, the number of coincident connections κ_{train} of a KC (with K dendrites) is binomially distributed as:

$$\kappa_{train} \sim Binom(K, \frac{X}{T}) \tag{S3}$$

Similarly the number of coincident connections κ_{test} in response to the test image (of a KC with K dendrites) is binomially distributed as:

$$\kappa_{test} \sim Binom(K, \frac{Y}{T}) \tag{S4}$$

If we have prior knowledge that a KC fired in response to the train shape, we know it must have a significantly over-average number of connections κ_{train} in region X, which shifts the distribution of κ_{test} upwards. In particular, the number of connections κ_{train} the cell makes in set X must be about 2 standard deviations (of the training population distribution) over the population mean (if $p = 0.05$). Mathematically:

$$\kappa_{train} \geq \frac{KX}{T} + 2\sqrt{\frac{KX}{T}\left(1 - \frac{X}{T}\right)} = K_X \tag{S5}$$

If we make the simplifying assumption κ_{train} is exactly on the boundary of the above inequality (which we denote K_X), the conditional distribution for κ_{test} (given that the cell fired in training) can be expressed as:

$$\kappa_{test}|\kappa_{train} \sim Binom(K_X, \frac{Y}{X}) \tag{S6}$$

The conditional firing probability can then be expressed as:

$$\mathbb{P}\big(\mathrm{KC}_{\mathrm{L}}^{\mathrm{Test}} = 1 \mid \mathrm{KC}_{\mathrm{L}}^{\mathrm{Train}} = 1\big) \approx \int_{\frac{KY}{T}+2\sqrt{\frac{KY}{T}\left(1-\frac{Y}{T}\right)}} Binom(K_X, \frac{Y}{X})(r)dr \tag{S7}$$

The above formula is hard to compute in closed form. However, it is sufficient to notice it has a strong dependence on how much the prior knowledge shifts the distribution of κ_{test} upwards. As a measure of upwards shift we consider the normalised difference between the means of the distributions for $\kappa_{test}|\kappa_{train}$ and κ_{test}. That is:

$$shift = \frac{\mathbb{E}\left[\kappa_{test}|\kappa_{train}\right] - \mathbb{E}\left[\kappa_{test}\right]}{\sigma\left[\kappa_{test}\right]} \tag{S8}$$

$$= \frac{1}{\sigma\left[\kappa_{test}\right]}\left[\frac{K_X Y}{X} - \frac{KY}{T}\right] \tag{S9}$$

$$= \frac{1}{\sigma\left[\kappa_{test}\right]}\left[\frac{KX}{T} + 2\sqrt{\frac{KX}{T}\left(1-\frac{X}{T}\right)}\right]\frac{Y}{X} - \frac{1}{\sigma\left[\kappa_{test}\right]}\frac{KY}{T} \tag{S10}$$

$$= \frac{2}{\sigma\left[\kappa_{test}\right]}\frac{Y}{X}\sqrt{\frac{KX}{T}\left(1-\frac{X}{T}\right)} \tag{S11}$$

$$= 2\frac{Y}{X}\frac{\sqrt{\frac{KX}{T}\left(1-\frac{X}{T}\right)}}{\sqrt{\frac{KY}{T}\left(1-\frac{Y}{T}\right)}} \tag{S12}$$

Assuming the base rates X/T and Y/T are small enough (modelling assumption), we can approximate the expression above to first order as shown in (S13). This assumption is biologically plausible, because the shapes considered do not cover the whole visual field of the ants, justifying an assumed sparse enough activation at vPN level.

$$shift \approx 2\frac{Y}{X}\frac{\sqrt{X}}{\sqrt{Y}} = 2\sqrt{\frac{Y}{X}} = 2\sqrt{\gamma_L} \tag{S13}$$

By manipulating (S8), we have shown how the shift (approximately) depends only on the ratio $Y/X = \gamma_L$. The same calculation can be conducted in the right hemisphere to show that the shift there depends only on $Z/W = \gamma_R$. Because γ_L and γ_R are linearly constrained via:

$$\gamma_L X + \gamma_R W = Y + Z \tag{S14}$$

there is a tradeoff between the two, increasing one necessarily decreases the other. We have shown the shifts equate across hemispheres when $\gamma_L = \gamma_R$, which justifies why that is also approximately where conditional firing probabilities equate. This in turn implies that $\gamma_L = \gamma_R$ is also approximately where left and right novelties equate.
