# Supplementary material for: Lateralised memory networks may explain the use of higher-order visual features in navigating insects
Source: PLoS Comput Biol. 2025 Jun 23;21(6):e1012670. doi: 10.1371/journal.pcbi.1012670 (PMC12225813; doi:10.1371/journal.pcbi.1012670)
Supplement: Attachment — Submitted filename: plos-bilateral-comments.docx [file pcbi.1012670.s006.docx]

## **Main comments:**

This work (a) implements a neural network model that emulates insect Mushroom Body (MB) in heading correction based on learned visual cues, (b) verifies the model against the real experiment data from Lent et al (2013a), and (c) argues for the proposition that insects use higher-order visual features in navigation. I recommend major revision, because the work presents comprehensive and interesting results and discussions regarding (a) and (b), but the reasoning for (c) is not compelling enough.

While the model shares many features with existing MB models, e.g. PN-KC-MBON layers, lateral inhibition amongst KCs by APL, the work focusses on the MB’s bilateral structure (which is relatively underexplored in literature). In particular, the investigation into the two parameters, crossover and overlap, (by computational modelling) is significantly valuable, as their values cannot be easily estimated or manipulated experimentally for real insects. Thus, I would like to read **more detailed discussions on them and other parameters, e.g., expanding line 103-116, line 185-188**.

However, the arguments for segmentation and those more general ones for FPM appear weak. Firstly, multiple tentative explanations are verbally described, rather than being tested, and at least some of them can be tested without modifying the model significantly. For example, line 227 says the data of Colony 2 might be explained by memory biased towards the first few views -- a simplest proof-of-concept test would be training the model using the first 1-10 images along the training route, instead of the total 30 images.

Secondly, and more importantly, most of the results of the computational model are verified statistically at the behavioural level (against real ant data). This approach hardly demonstrate whether insects or the model use higher-order visual features (FPM or segmentation). If the work’s methodology is primarily performing verification against real experiments (and by so supporting Lent et al (2013a)’s proposition), then the work would probably benefit more from the detailed discussions (requested above) to show that, in general, insects use FPM and segmentation, as long as they share the generic MB structure and the vision-to-MB pathway. It would be much more convincing, if some (subsets of) neurons in the model could be identified as a particular neural representation of FPM or segmentation. A theoretical approach like the analysis for nested shapes (from line 128) might also be considered. However, the current analysis only covers a very special case, which is a good start for the arguments regarding FPM, but not mentioning segmentation at all.

The theoretical analysis, especially in line 146-175, is also hard to comprehend. The equations and explanations would read more straightforward, if the expectation and the probability can be written explicitly in terms of W, X, Y, Z. If the rigorous mathematical deduction is not accessible (not really necessary for a computational manuscript anyway), some stats and plots (like those for the insensitivity of parameters in line 176-188) might be easier for readers to understand. Given the winners-take-all mechanism in the KC layer, alpha seems incorrect in Equations (3) and (4).

Overall, the manuscript presents a valid model and explores its behaviours in a comprehensive and insightful manner. From a computational modelling perspective, it would be ideal if the test could be performed dynamically, i.e. the agent updates its movements and visual views successively, instead of N_test=1, but I think this request is somewhat redundant, as I haven’t seen evidence showing potential behavioural differences between these two cases (at least when there are no obstacles). However, I’m not persuaded by the manuscript that the model behaviours demonstrate ‘the use of higher-order visual features’ as claimed by the title.

Below I list a few points that I’d like to receive clarification. I can see some points might affect my comments above, but I hope I haven’t misunderstood the authors in any significant way.

## **Need clarification:**

Line 51: Why use ‘However’? Is this ‘bias’ inspired by real neuroanatomy or not?

Line 55: Does ‘parameter values’ refer to FPM’s values or other parameters’ values?

Line 91: Can more justification be provided for this assumed ‘equal importance’ between low novelty signal and lateral balance signal? Is it because the same beta=4 used for both signals (as in section Generating Heading Distributions from line 493)?

Line 99: Can more justification be provided for this distance between modes as a measure of performance?

Line 407: It’s not clear what theta’ and phi’ are.

Line 500: Does this P(S, delta) need to be normalised to be a proper ‘probability’?

Line 528: Is this KDE smoothing really necessary?

Line 530: Please use a different notation for this point, as x is used for pixel/vPN.

## **Formatting issues:**

Spelling consistency, e.g. ‘memorization’ in line 8 vs. ‘organised’ in line 9, ‘centre’ vs ‘center’.

Change ‘it’s’ to ‘its’.

‘Train image’ or ‘training image’.

Use their abbreviations instead of the full terms after the first appearance. VPN used in Fig 3, but vPN in text.

Capitalisation of subsections is inconsistent in Methods.

Units, e.g. m in line 393, should not be italic. If the manuscript is prepared in LaTeX, the siunitx package might be handy.

## **Minor suggestions:**

Line 3: Remove ‘homing and’, as ‘homing’ is repeated in line 5.

Line 27: Insert ‘a’ after ‘at’.

Line 41: Move ‘in navigating wood ants’ after ‘segmentation)’ in line 42.

Line 49: Change single quotation marks on ‘eyes’ to double quotation marks for consistency.

Line 88: Mention tropotaxis.

Line 134: Make it explicit that here the test shape must be rectangle.

Line 326: This section may benefit from discussing how assumed model features, e.g., winners-take-all in the KC layer, might contribute to attention-like mechanism.

Line 401: This part might benefit from adding some notations (of points and angles) in Fig 1.

Line 409: Change ‘black and white’ to ‘greyscale’.

Line 438: Change w_xkc to w_xKC (as for other subscripts).

Line 458: Change w_x to w_xKC.

Line 480: Add perhaps some super/sub-scripts to differentiate the two w_KC before/after training.

Line 535: Add ‘DBM’ after ‘modes”)’, as DBM is used in the following equation.
