# Supplementary material for: Lateralised memory networks may explain the use of higher-order visual features in navigating insects
Source: PLoS Comput Biol. 2025 Jun 23;21(6):e1012670. doi: 10.1371/journal.pcbi.1012670 (PMC12225813; doi:10.1371/journal.pcbi.1012670)
Supplement: Attachment — Submitted filename: Response to Reviewers.docx [file pcbi.1012670.s007.docx]

**Reviewer #1:**

**This study showcases an elegant algorithm that lays out, how a processing of higher visual features may emerge by considering the hemispherical structure of the insect brain/mushroom bodies. Within the context of visual navigation in ants, this lays an important step considering visual features other than the holistic usage of the entire panorama.**

--We thank Reviewer #1 for their positive and constructive comments

- **Please add a more detailed description of your code to the readme.**

*–done.*

- **LL. 20-25: Just for the context within the range of naturally occuring behaviours in ants, where does the Lent study fit into the foraging process? Would this be mostly relevant for a "final approach", where the ant has almost reached the feeder and wants to find the very specific location? Or would this FPM also reflect a mechanism which could be applied at any section during route following, from the nest to the food location?**

*–The results of Lent et al (2013a) could in principle be relevant for any part of a visual-memory guided route as it relates to the general problem of setting direction. Of course, a direct analogy to the experimental results would be when a large object / shape is present in the frontal part of the visual scene, which in natural settings could be a bush / tree or for a more distant panorama could be a hill / mountain. [We have audited the discussion to make the connection to natural navigation clearer (edited manuscript l.401-403)].*

- **LL. 191-198: The test shapes cannot be contained in the train shapes, because they do not have the same relative shape? For example, the Scalene shape triangle in 4B would only be contained in the train shape if it were, for example, scaled down by 20% in the x and y?**

*–Having the test shape contained within the train shape is an assumption that simplifies the theoretical analysis (if the train shape is contained within the test shape, the same theoretical analysis can also be conducted). Whilst it may seem restrictive at first, it is actually what is likely to happen in natural settings, for instance if we observe the same shape from two different distances, then (as is correctly noted in the review), having the same relative shape means that there will be containment. [We have clarified that this is a simplifying assumption for that part of the analysis (edited manuscript l.137-141)].*

- **LL. 327-336: Attention in Human EEG studies show increased activity in the hemisphere associated with the processing of the task relevant stimulus (Antonov et al 2020, "Too little, too late, and in the wrong place..."). In the context of this model, can you imagine a mechanism which up-/downregulates the processing of the task relevant segments?**

*–This is an interesting observation and connection to human neuroscience. In the context of insect neuroscience, visual selective attention has been demonstrated for tracking salient objects (e.g. Wiederman and O’Carroll, 2013). Such attentional mechanisms could downregulate the neural responses of some visual features in favour of others (favoring some task relevant information), and this regulation could be hemispheric. However, it remains unknown if such attentional mechanisms play a part in the visual representations used in navigation, and that is definitely something that warrants further research. [We briefly mention this in the context of discussion of segmentation (manuscript l.347-349)].*

- **Furthermore, in visually complex scenes there will be many more shapes in the visual fields. Would this mechanism work with learnt shapes covering the entire panorama, or would this need some selective attention mechanism which depresses the computation of non-relevant shapes?**

*–For a feature like the FPM to be used as part of navigation, some shape has to be defined over which the FPM is computed. In the context of our own work / interpretation, the emergence of the FPM from a bilateral memory network would still require that some shape be defined. So indeed a selective attention (or higher attribution of projections to a central part of the visual field) are candidate mechanisms for the extraction of a task relevant segment. [As above, we briefly mention this in the context of discussion of segmentation (manuscript l.350-356)].*

- **L. 388: Maybe change "view" to "panoramic view"? So that the distinction between monocular and binocular is stronger.**

*–done.*

- **L. 436: Why Nkc = 25,000? In total 50,000. Other studies use less KCs for representing the whole MB. If you used similarly lower numbers, would that negatively effect the results of your model, and if so, how?**

*–The number of Kenyon Cells we chose to use is representative of the numbers found in insects such as ants and bees. However the analysis would work analogously for any value Nkc, that is if it is sufficiently large (1000 would definitely be sufficient for instance) for law of large number statistics to apply. [We have added a brief note to this effect in the methods (edited manuscript l.453-454)].*

- **L. 455: The crossover probability covers the entire contralateral vPN space, or only the space minus overlap?**

*–The entire contralateral vPN space (“eye”) has its own projections to the ipsilateral and contralateral MBs (according to the crossover probability parameter). So the overlap region is represented twice. [We have checked the phrasing of this section to be clear (edited manuscript l.472)].*

- **L. 514: The crossover is from the contralateral "eye"? In other words, 0-50% random pixels from the contralateral retina?**

*–The crossover denotes the probability (ranging from 0 to 0.5) that a vPN connection is created from one eye to the contralateral MB. In essence it is a measure of ipsilateral bias in projections to the Mushroom Bodies (with 0 being a maximal amount of ipsilateral bias).[We have checked the phrasing of this section to be clear (edited manuscript l.472)].*

- **S1 Fig: Above the plots, maybe show the training image like in S3.**

*–We have updated the figure and caption so that it also displays the train and test images (edited manuscript S1 Fig A,B).*

- **And lastly, i want to recapitulate the processing steps as i understood them; due to the manuscript-structure, the individual, but consecutive steps were a little too distributed for me. If there is anything missing/wrong in my recapitulation, maybe point out these steps more in detail.**

1. **Generation of images from the centre of the scene moving straight towards the feeder, so that the feeder would always be in the centre/origin of the image. These images represent the route memories.**
2. **Training the MB-Model using the route memories.**
3. **Presenting test-images to the model with different rotations, so that a novelty function for (most of) the panorama can be generated. For each shape, were these also presented from different places on the route, just like route memories?**
4. **Applying the Monte Carlo method to generate the most likely heading directions, dependent on the novelty sum and novelty difference.**
5. **Comparison with the behavioural data.**

*–Thank you for taking the time to clarify this and it is an accurate description of the steps used to generate distributions of heading directions and compare them to behavioural data, except for a minor caveat. That is that steps (2-4) are repeated for 30 crosspairs of parameter values with 50 model instantiations each, which results in 50*30 = 1500 repetitions of the estimation process that are pooled together. The pooling is conducted to show the aggregate behaviour of the model over a broad range of parameter values and random instantiations of weights, rather than the results for one set of parameter values. [We have audited the relevant sections to make sure it is clear (manuscript l.536-543)].*

**Reviewer #2:**

**This work (a) implements a neural network model that emulates insect Mushroom Body (MB) in heading correction based on learned visual cues, (b) verifies the model against the real experiment data from Lent et al (2013a), and (c) argues for the proposition that insects use higher-order visual features in navigation. I recommend major revision, because the work presents comprehensive and interesting results and discussions regarding (a) and (b), but the reasoning for (c) is not compelling enough.**

*–We thank the reviewer for clarifying the key points of the paper, and apologise if there are aspects of this study which are unclear. Given that our work relies on behavioural data, our purpose is not to show that ants create and memorise a neural representation of the FPM, but rather that behaviours consistent with the use of such features could emerge from simple anatomically constrained networks (manuscript l.40-44). Indeed, in our interpretation, the “FPM” is not encoded in any part of the neural network, but exists within the aggregate properties of a (bilateral) trained network and how these interact with a novel test shape. In consequence of this, we are currently not able to comment on whether ants memorise and use a representation of the FPM. [We have changed some of the phrasings in the main text to clarify this (edited manuscript Introduction l.7,9,14 / Author Summary l.5,8 / Main Text l.41, l.249, l.267, l.296, l.311, l.338, l.401)].*

**While the model shares many features with existing MB models, e.g. PN-KC-MBON layers, lateral inhibition amongst KCs by APL, the work focusses on the MB’s bilateral structure (which is relatively underexplored in literature). In particular, the investigation into the two parameters, crossover and overlap, (by computational modelling) is significantly valuable, as their values cannot be easily estimated or manipulated experimentally for real insects. Thus, I would like to read more detailed discussions on them and other parameters, e.g., expanding line 103-116, line 185-188.**

*–We thank the reviewer for this observation. Indeed the crossover and overlap parameters have not been (extensively) studied previously, although overlap is studied in (Steinbeck et al, 2024). For the purposes of our own study, scanning different values of the parameters is merely a way to show the stability (robustness) of our model results against parameter perturbations. That is also why we pool model runs over different (biologically plausible) values of the crossover and overlap parameters, to show that the aggregate behaviour of the model is still a good fit for the biological data. [We have changed some of the phrasings in the main text to clarify this (edited manuscript l.194-197)]*

**However, the arguments for segmentation and those more general ones for FPM appear weak. Firstly, multiple tentative explanations are verbally described, rather than being tested, and at least some of them can be tested without modifying the model significantly. For example, line 227 says the data of Colony 2 might be explained by memory biased towards the first few views -- a simplest proof-of-concept test would be training the model using the first 1-10 images along the training route, instead of the total 30 images.**

*–We have added a supplemental figure (S4 Fig) and updated the text to better explain how the length of the training path changes model outputs. The outputs of the computational model further corroborate our hypothesis that a model with memories biased towards the beginning of the route has the quality of left and right memories balancing precisely at the peak-defined direction. [We have added a paragraph to better explain this (edited manuscript l.231-242)]*

**Secondly, and more importantly, most of the results of the computational model are verified statistically at the behavioural level (against real ant data). This approach hardly demonstrate whether insects or the model use higher-order visual features (FPM or segmentation). If the work’s methodology is primarily performing verification against real experiments (and by so supporting Lent et al (2013a)’s proposition), then the work would probably benefit more from the detailed discussions (requested above) to show that, in general, insects use FPM and segmentation, as long as they share the generic MB structure and the vision-to-MB pathway. It would be much more convincing, if some (subsets of) neurons in the model could be identified as a particular neural representation of FPM or segmentation. A theoretical approach like the analysis for nested shapes (from line 128) might also be considered. However, the current analysis only covers a very special case, which is a good start for the arguments regarding FPM, but not mentioning segmentation at all.**

*–We thank the reviewer once again for this clarification. As mentioned above, it is correct to point out that we are studying behavioural data, and hence we are not able to comment on the use of features such as FPM, or the use of segmentation. To clarify, the major purpose of this paper is to show how behaviour consistent with the use of such features might emerge from the known neuroarchitecture of the insect memory centre and its interaction with simple visual scenes (original manuscript l.40-44). In so doing, we answer the open question as to where these features could be stored by claiming they are stored in aggregate network properties and their interaction with scenes. Regarding segmentation, our model does not achieve a perfect fit for the biological data, so any conclusion is only tentative / speculative (different interpretations that could explain this behavioural data are explored in the Discussion section). [We have changed some of the phrasings in the main text to clarify these points. edited manuscript l.41, l.249, l.267, l.296, l.311, l.338, l.401, l.249, l.259, l.267)]*

**The theoretical analysis, especially in line 146-175, is also hard to comprehend. The equations and explanations would read more straightforward, if the expectation and the probability can be written explicitly in terms of W, X, Y, Z. If the rigorous mathematical deduction is not accessible (not really necessary for a computational manuscript anyway), some stats and plots (like those for the insensitivity of parameters in line 176-188) might be easier for readers to understand. Given the winners-take-all mechanism in the KC layer, alpha seems incorrect in Equations (3) and (4).**

*–Thank you for this constructive criticism and it is important for us to provide the maths in a way that is useful to different types of reader. We have simplified this part of the main text and outsourced more detailed aspects to the supplemental (S5 Appendix). In particular, we have removed unnecessary parameters (such as the learning rate alpha) from the expressions in the main text (edited manuscript l.154), retaining only what is needed to explain the key points of the argument. We hope this makes it more readable. [We have also added a supplemental S5 Appendix to better explain some aspects of the theoretical findings].*

**Overall, the manuscript presents a valid model and explores its behaviours in a comprehensive and insightful manner. From a computational modelling perspective, it would be ideal if the test could be performed dynamically, i.e. the agent updates its movements and visual views successively, instead of N_test=1, but I think this request is somewhat redundant, as I haven’t seen evidence showing potential behavioural differences between these two cases (at least when there are no obstacles). However, I’m not persuaded by the manuscript that the model behaviours demonstrate ‘the use of higher-order visual features’ as claimed by the title.**

*–As we have discussed above, the original paper used the behavioural data from ants to propose that visual navigation might rely on higher-order visual processing (manuscript l.40-44). By building a bottom-up model, we are hoping to show that the observed ant behaviours can emerge from the interaction of the basic neural architecture, a simple behavioural task and the details of the visual patterns. Thus we aren’t trying to prove that ants use higher-order visual features, so much as showing that behaviours that can be interpreted that way, emerge naturally from this basic architecture. [We have audited, and made some changes to, the title, abstract and discussion to try and make this clear.]*

**Need clarification:**

**Line 51: Why use ‘However’? Is this ‘bias’ inspired by real neuroanatomy or not?**

*–Given that there is no study in the literature on the proportion of ipsilateral versus contralateral visual projections into the MBs (to our knowledge), this constitutes a modelling assumption. [We have made this clearer in the text (edited manuscript l.51)]*

**Line 55: Does ‘parameter values’ refer to FPM’s values or other parameters’ values?**

*–We are here referring to the model’s parameter values (alpha, K, p, crossover, overlap) but we could equally be referring to the specific value of the FPM, and to certain weight instantiation parameters (constant or random weights). [We have made this clearer in the text (edited manuscript l.55)]*

**Line 91: Can more justification be provided for this assumed ‘equal importance’ between low novelty signal and lateral balance signal? Is it because the same beta=4 used for both signals (as in section Generating Heading Distributions from line 493)?**

*–This is more of a modelling choice rather than an empirically deduced observation. The reasoning is that in the absence of a way to assess which is more important, we assumed both are equally important as part of our modelling procedures. Using beta=4 for both signals is the way this modelling decision translates into the mathematical formula for probability of acceptance. [We have made this clearer in the text (edited manuscript l.90-91)]*

**Line 99: Can more justification be provided for this distance between modes as a measure of performance?**

*–The location of the major mode within the data was the statistic studied in the original work of Lent et al (2013a). Given that the experimental distributions are often very centered around one angle, the major mode is considered the “true” goal directions of the ants and the variance represents noise around that direction that could come from various sources. By following this line of reasoning, we use the distance between modes to quantify the quality of our predictions. [We have made this clearer in the text (edited manuscript l.98-101)]*

**Line 407: It’s not clear what theta’ and phi’ are.**

*–We have added a depiction of the stimulus angles theta and phi in the schematic of Fig 1 (orange lines and angles) [We have also updated the Fig 1 legend accordingly].*

**Line 500: Does this P(S, delta) need to be normalised to be a proper ‘probability’?**

*–The formula for P(S,Delta) could be normalised over all possible directions, but it does not need to be normalised for the algorithm to function. Because directions are generated at random (Monte Carlo method), it is only the relative acceptance probabilities that matter. The Monte Carlo is useful here because we pool together the tentative directions for many model instantiations and parameter settings, which means that a “closed form” for the distribution is not accessible. [We have audited the text for clarity]*

**Line 528: Is this KDE smoothing really necessary?**

*–The KDE smoothing is important to estimate mode locations in non-unimodal distributions. That is because the frequency of a given bin could be greater than its neighbours without being a “true” mode of the distributions. So smoothing is needed to filter out the “fake” modes. [We have made this clearer in the text (edited manuscript l.546)]*

**Line 530: Please use a different notation for this point, as x is used for pixel/vPN.**

*–done*

**Formatting issues:**

**Spelling consistency, e.g. ‘memorization’ in line 8 vs. ‘organised’ in line 9, ‘centre’ vs ‘center’.**

*–We changed these so as to consistently use British english.*

**Change ‘it’s’ to ‘its’.**

*–done*

**‘Train image’ or ‘training image’.**

*–We now consistently use train image.*

**Use their abbreviations instead of the full terms after the first appearance. VPN used in Fig 3, but vPN in text.**

*–We changed the figure so that we consistently use vPN.*

**Capitalisation of subsections is inconsistent in Methods.**

*–We changed the capitalisation so that only the first word is capitalised.*

**Units, e.g. m in line 393, should not be italic. If the manuscript is prepared in LaTeX, the siunitx package might be handy.**

*–Done with siunitx.*

**Minor suggestions:**

**Line 3: Remove ‘homing and’, as ‘homing’ is repeated in line 5.**

*–done*

**Line 27: Insert ‘a’ after ‘at’.**

*–done*

**Line 41: Move ‘in navigating wood ants’ after ‘segmentation)’ in line 42.**

*–done*

**Line 49: Change single quotation marks on ‘eyes’ to double quotation marks for consistency.**

*–done*

**Line 88: Mention tropotaxis.**

*–Thank you for bringing this to our attention. To our understanding, tropotaxis would refer to the scenario where a difference in bilateral signals is used to orient towards or away from a source. Given that our modelling approach uses a balancing of bilateral signals, we deemed that it is not directly related to tropotaxis.*

**Line 134: Make it explicit that here the test shape must be rectangle.**

*–Whilst we use rectangles as default shapes in schematics, the train and test shapes do not have to be rectangles for this analysis to work. The arguments work for any test shape contained within the train shape, and can be easily adapted to the case where the train shape is contained within the test shape. [We have made this clearer in the text (edited manuscript l.140-141)]*

**Line 326: This section may benefit from discussing how assumed model features, e.g., winners-take-all in the KC layer, might contribute to attention-like mechanism.**

*–The major contribution of our model to explaining the apparent segmentation/attention-like mechanism is the effect of balancing left and right novelties. It is often the case that balancing left and right novelties shifts and changes the location of predicted modes in a way that brings them closer to the experimental results, but the fit in these cases is not as good as that of the previous experiments, making any conclusions speculative / tentative (we consider various options in the Discussion). [We have audited the discussion to hopefully make this clearer (edited manuscript l.272-275, l.254-256)]*

**Line 401: This part might benefit from adding some notations (of points and angles) in Fig 1.**

*–We added notation showing the angles phi and theta in Fig 1A, which should help make this section clearer. (edited manuscript Fig 1A and l.421)*

**Line 409: Change ‘black and white’ to ‘greyscale’.**

*–done*

**Line 438: Change w_xkc to w_xKC (as for other subscripts).**

*–done*

**Line 458: Change w_x to w_xKC.**

*–done*

**Line 480: Add perhaps some super/sub-scripts to differentiate the two w_KC before/after training.**

*–done*

**Line 535: Add ‘DBM’ after ‘modes”)’, as DBM is used in the following equation.**

*–done*

**Reviewer #3:**

**The authors revisit here the question of whether ants are able to use one specific higher-order visual feature, the "fractional position of mass" (FPM), to navigate. While this may seem like a rather niche or narrow question, the methodology shown in this work and its general message are very significant to the field: By applying a model constrained by the known anatomy and visual information processing in the ant brain, the authors demonstrate that encoding and recalling of an otherwise thought "higher-order" kind of information can actually be a purely emergent property of the brain's bilateral organization, rather than a specifically tuned, dedicated module. Their model reproduces the experimental findings from the Lent et al 2013 paper quite remarkably. I also appreciate that the authors contextualize their model of the Mushroom Bodies within the broader context of areas involved in navigation, the Central Complex and Lateral Accessory Lobes (paralleling the recently uncovered role of PFL2 cells in Drosophila). The approach shown in this paper and its main conclusion (that "a bilateral brain architecture allows ants to balance the quality of their memories in left and right hemispheres when navigating") clearly fit whithin the current state of research in the neurobiology of insect navigation, and represent in my opinion an overall coherent and convincing example of how modelling allows us to draw the link between complex behaviour and brain connectivity, making this work a strong contribution to the field.**

--We thank the reviewer for their positive perspective on the paper.

**Some comments for the authors:**

- **"However, if the memory of the path were biased towards the first few views (due to some unknown mechanism), then we would expect that left and right novelties equate at the peak-defined direction, providing a tentative explanation for the bifurcation in colony behaviors." (l. 226 - 229)**

*–We have added a supplemental figure (S4 Fig) and updated the text to better explain how the length of the training path changes model outputs. The outputs of the computational model further corroborate our hypothesis that a model with memories biased towards the beginning of the route has the quality of left and right memories balancing precisely at the peak-defined direction. [We have added a supplemental and paragraph to better explain this (edited manuscript l.231-242)]*

- **It would maybe be beneficial to quantify the change in information received by the ommaditia between two steps in the simulation, as one would expect a stronger change in the signal between two steps when the angular size of the object is greater (i.e. a smaller change in the familiarity between two steps at the start of the route compared to the end).**

*–This is an interesting point. Indeed the shape would change more the closer the ant is to the wall. However it is important to note that the experimental setup was such that these changes are subtle, as studied in the Supplementals of Lent et al (2013a). Still, subtle changes in the shape over a 0.6m path can lead to qualitative changes in the results of the model. To explore this further within the paper, we have added a Supplemental figure (S4 Fig) explaining the difference in results for 4B between training on the path and training on the first image. Amongst other things, this shows how the shape changes with distance from the centre of the arena. [We have added a supplemental to explore this S4 Fig]*

- **Also, including some model results using more complex visual scenes and/or a more complex insect eye model might generate some very interesting predictions to be tested experimentally.**

*–Given that the current value of the modelling is through its connection to Lent et al, we wanted to be thorough with that comparison. Investigating complex visual scenes is a very interesting idea, but one that would require significant work and we think would definitely justify its own manuscript.*

*We appreciate that our approach relies upon the simplicity of the visual scene and we expect feature extraction within the ant’s visual system would alter our predictions in more complex scenes. Indeed we have already shown how the predictions are sensitive to the fine details, where with the reflected triangle example we show there can be different possible behaviours depending on whether the learning is biased towards the beginning of the route or the end of it. This type of idea can be tested experimentally by tuning the path length prior to reaching the feeder and provided the model predictions are correct, this also opens up avenues for studying how views are weighted in memory based on their temporal distance from reward (trace conditioning).*
